# Data on the influence of dynamical impact by mixing on characteristics of turbulent flows in circular pipes

**DOI:** 10.1016/j.dib.2022.108276

**Published:** 2022-05-17

**Authors:** Andrei Miheev

**Affiliations:** Institute of Power Engineering and Advanced Technologies, FRC Kazan Scientific Center of RAS, Russia

**Keywords:** Flow structure, Turbulent characteristics, Dynamical impact, Circular pipe

## Abstract

The article presents experimental data on the kinematic structure of flows in circular channels in conditions of dynamical impact on the flow. The experimental data on the influence of dynamical impact by mixing on flow parameters were obtained. Averaged turbulent characteristics were measured at different rotation velocities of mixers and at different distances from them. Optical measurements yielded the profiles of velocities and turbulent characteristics of flows plotted in representative cross sections behind the source of dynamical impacts.

To obtain new experimental data on the influence of dynamical impact by mixing on turbulent characteristics of flows, we measured the averaged profiles of streamwise and transverse velocity components, their RMS deviations, and vorticity in different cross sections of the channel (at the distance of 10 and 20 diameters from the system of mixers) at different rotation frequencies of mixers. The measured profiles were then compared with the profiles in undisturbed turbulent flows.

## Specifications Table

Every section of this table is mandatory. Please enter information in the right-hand column and remove all the instructions in gray, italicised text.

**Table:** 

Subject	Hydrodynamics
Specific subject area	Hydrodynamics (the effect of dynamical impacts on turbulent flows)
Type of data	Graph Figure Text File
How the data were acquired	The data were acquired by optical measurements of velocity fields. Smoke visualization images were processed using Smoke Image Velocimetry method, which provides estimation of the dynamics of velocity vector fields and vorticity fields based on frame-by-frame processing of high-speed video recordings. Vector fields of velocity in this method are estimated from the measured frame-to-frame displacements of turbulent structures visualized by smoke in the light sheet.
Data format	Raw Analyzed
Description of data collection	Special experimental facility was designed to study the influence of the dynamical impact on gas flows. The facility is a transparent circular channel made of polycarbonate. Flow rate through the channel is generated by a blower operating in a suction mode. A working fluid is the air from the receiver. The flow velocity, and hence the Reynolds number is controlled by a set of calibrated critical flow nozzles. Four small mixers are installed on a support inside the pipe. Special tracer particles were introduced to the flow using an aerosol generator mounted upstream of the inlet to the test section. The flow rate of these particles was adjusted by a special regulator. The light sheet employed for flow visualization was generated by a continuous laser.
Data source location	Institution: Institute of Power Engineering and Advanced Technologies, FRC Kazan Scientific Center, Russian Academy of Sciences City/Town/Region: Kazan Country: Russia
Data accessibility	Repository: Mendeley data Title of the dataset: Data on the influence of dynamical impact by mixing on characteristics of turbulent flows in circular pipes DOI: 10.17632/9jvjg48d38.1 URL: https://data.mendeley.com/datasets/9jvjg48d38/1

## Value of the Data

• The obtained data are useful for complex development of the fundamental basis for new control methods of transport processes by dynamical impacts in turbulent flows.
• Besides, provided that further research into different ways of dynamical impacts on flows will be carried out, the obtained data will contribute to formulation of recommendations for the development of new flow control technology, e.g. drag reduction.
• The obtained data can be employed for the development of cutting-edge numerical simulation methods owing to the novel reliable information on the characteristics of different complex types of flows.

## 1 Data Description

The data describe the influence of dynamical impact on the kinematic structure of turbulent flows in circular channels. Experimental data (profiles of velocities and turbulent characteristics behind the source of dynamical impact) were obtained for different cross sections along the channel length. These data were obtained for one magnitude of the flow rate.

Prior to main experiments, to estimate the parameters of undisturbed flow, the profiles of turbulent statistics were measured without the source of dynamical impact. Measurements were performed at the distance of 64d from the inlet. The system of mixers was mounted in this cross section in subsequent measurements. The obtained experimental data are in fairly good agreement with the known measurements in turbulent flows. Additionally, to improve the validity of comparison, we carried out the experiments with the system of mixers in place but not activated.

**Fig. 1:**
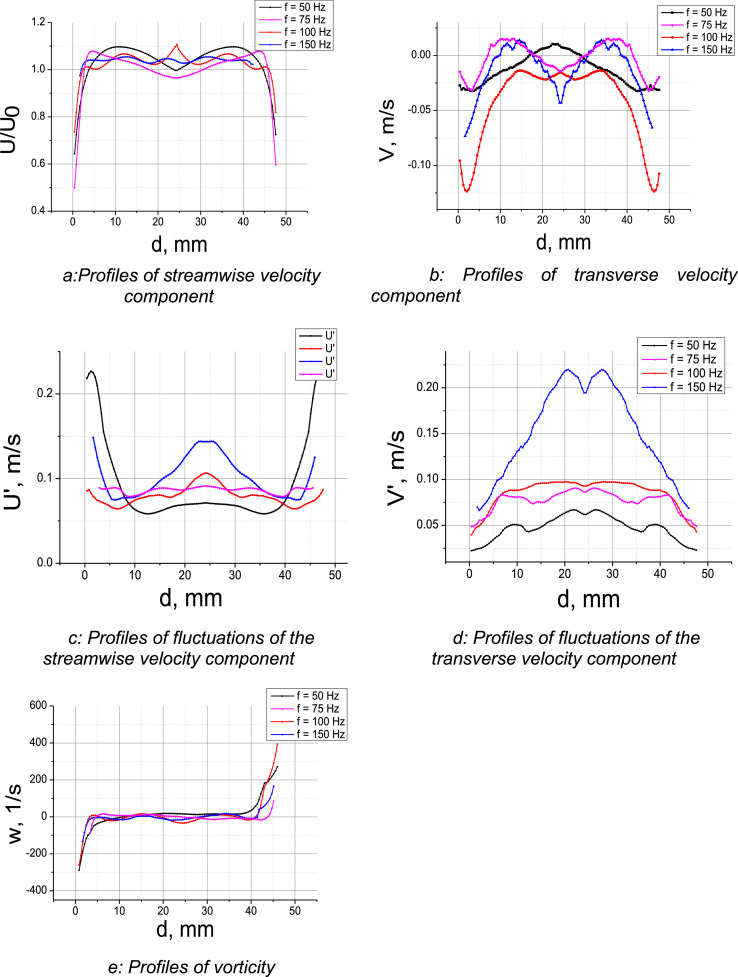
(1a): Profiles of streamwise velocity component (1b): Profiles of transverse velocity component (1c): Profiles of fluctuations of the streamwise velocity component (1d): Profiles of fluctuations of the transverse velocity component (1e): Profiles of vorticity

Then, in order to obtain new experimental data on the influence of dynamical impact by mixing on turbulent characteristics, we measured the averaged profiles of the transverse component of velocity, its RMS deviations, as well as vorticity in different cross sections of the channel (at the distance of 10 and 20 diameters from the system of mixers) and for different rotation frequencies of the mixers (f_mixers_ = 50; 75; 100; 150 Hz). The Reynolds number in experiments was *Re* = 4000. Fig. 1 demonstrates the profiles of averaged characteristics of flow behind the source of dynamical impact in the form of a system of mixers at the distance of 10 d from them.

Different profiles correspond to different rotation frequencies of mixers. The analysis of these profiles leads to the conclusion that introduction of this kind of dynamical impact into turbulent flows results in significant distortion of velocity profile compared to a classic profile typical of turbulent flow.

From the analysis of RMS deviations of streamwise and transverse velocity components, it is clear that the increase in the rotation frequency of mixers does not always lead to the increase in RMS deviation of velocity components.

The analysis of vorticity profiles shows that forced mixing of flow by symmetrically located mixers has no significant effect on vorticity compared to flows without mixing; it creates neither swirl nor thrust.

**Fig. 2:**
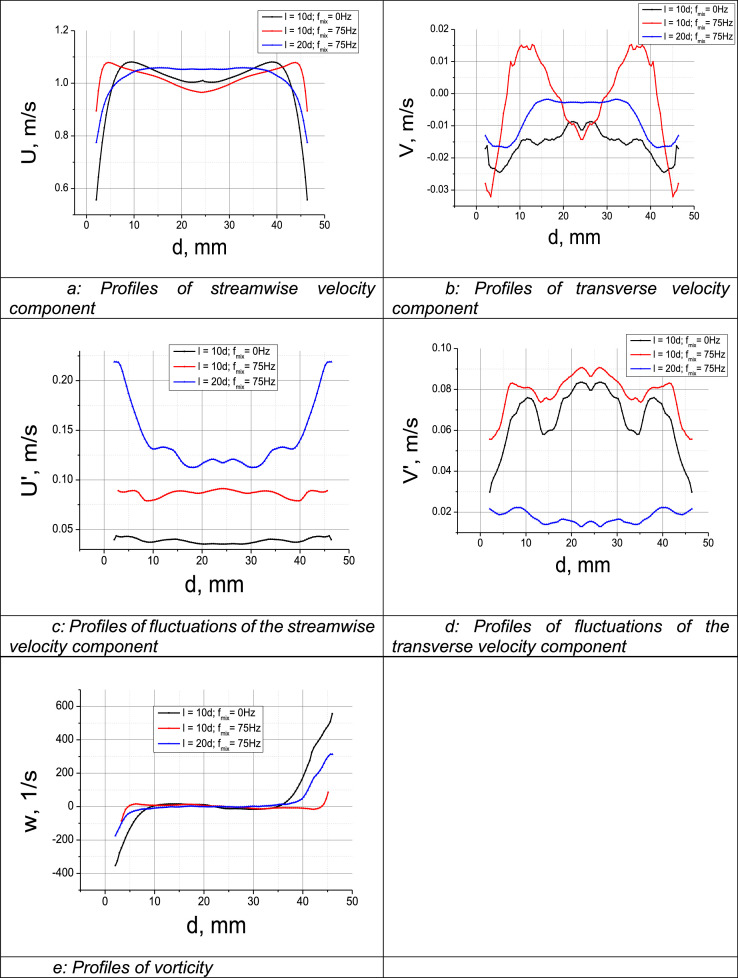
(2a): Profiles of streamwise velocity component (2b): Profiles of transverse velocity component (2c): Profiles of fluctuations of the streamwise velocity component (2d): Profiles of fluctuations of the transverse velocity component (2e): Profiles of vorticity

Then the evolution of profiles of turbulent statistics is analyzed depending on the distance to the system of mixers in one of the flow regimes (Fig 2).

Forced mixing at the distance of 10 diameters from the system of mixers has no considerable effect on the shape of streamwise velocity profile. It is almost identical to the profile in flows without mixing. At the distance of 20 diameters from the system of mixers, the differences from steady flows are significant. The profile shape in the near-wall region is somewhat similar to the laminar profile. However, its shape near the flow core is still close to turbulent.

The comparison between the profiles of RMS deviations of streamwise velocity component demonstrates that forced mixing of flow results in increased fluctuation at the distance of 10 diameters from the system of mixers and in even stronger fluctuation at the distance of 20 diameters. Interestingly, the highest values of RMS deviations of streamwise velocity are observed in the near-wall region.

Analyzing vorticity profiles, we came to the conclusion that forced mixing has no significant effect on vorticity compared to the case without missing and is almost independent of the distance to the source of dynamical impact.

## 2 Experimental Design, Materials and Methods

### 2.1 Materials

**Fig. 3:**
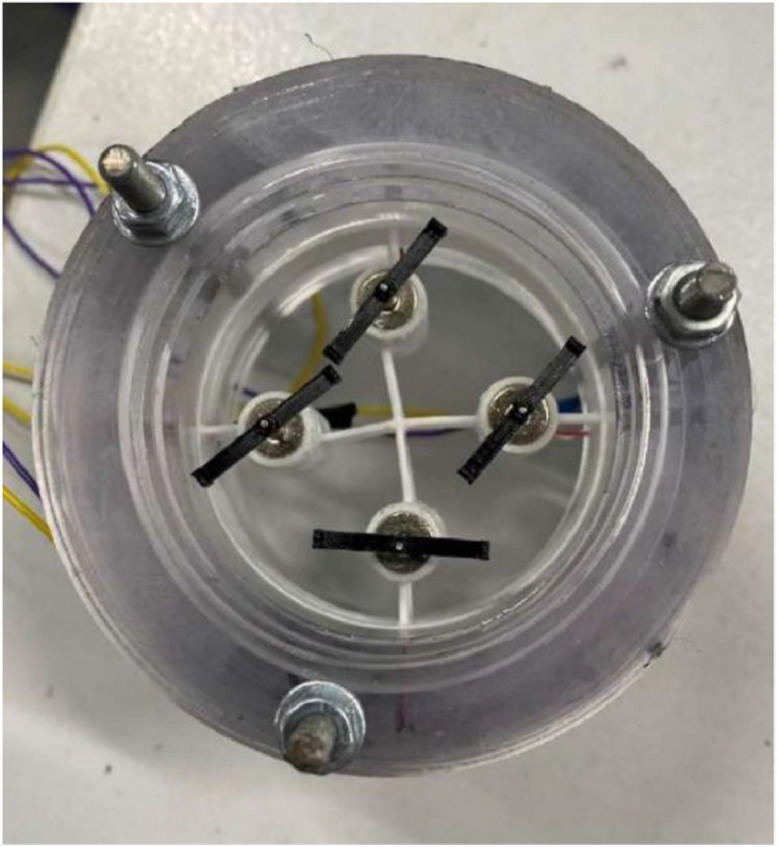
The source of dynamical impact on the flow (a system of mixers).

Special experimental facility was designed to study the influence of the dynamical impact on gas flows. The facility is a transparent circular channel made of polycarbonate with the internal diameter *d* = 47 mm and total length of 12 m consisting of 3 m long sections. Flow rate through the channel is generated by a blower operating in a suction mode. A working fluid is the air from the receiver. The flow velocity, and hence the Reynolds number (*Re* = UD / ν, where U is the average velocity, D – pipe diameter, ν – kinematic viscosity) is controlled by a set of calibrated critical flow nozzles. Four small mixers are installed on a support inside the pipe at the distance of 3 m from the inlet. The source of dynamic impact on the flow in the form of a mixer system is shown in Fig. 3.

The mixers are intended for introduction of disturbances to the flow without generation of motion or thrust. The rotation velocity of rotors is 50; 75; 100; 150 revolutions per second. Such a design is convenient for SIV measurements of instantaneous vector fields of velocity in planes illuminated by the laser sheet. Table, describing all used parameters is given below (Table 1).

**Table 1:** Experiment parameters.

Parametr	Value
Rotation velocity of rotors	50…150 Hz
Channel internal diameter	47 mm
Channel total length	12 m
*Re* number	4000

### 2.2 Method of investigation

For the purpose of flow visualization, special tracer particles were introduced to the flow using an aerosol generator mounted upstream of the inlet to the test section. The flow rate of these particles was adjusted by a special regulator. The light sheet employed for flow visualization was generated by a continuous laser. The light sheet thickness was 1 mm. Video recording was performed by a monochrome high-speed camera with the frequency of 4000 fps, which is an optimal frequency for subsequent analysis and processing of images at the maximum frame resolution of 1280 × 100 pix. Smoke visualization images were processed using SIV method [1], [2], [3], [4], [5], [6], which provides estimation of the dynamics of velocity vector fields and vorticity fields based on frame-by-frame processing of high-speed video recordings. Vector fields of velocity in this method are estimated from the measured frame-to-frame displacements of turbulent structures visualized by smoke in the light sheet. Smoke is introduced to the flow by commercial smoke generators (aerosol generators) that provide the particle size (droplet size) of about 1 µ and the smoke density close to the air density. Such particles closely follow velocity fluctuations with the frequency of up to 10 kHz in turbulent flows. When turbulent structures move almost without rotation, their displacements are measured as follows. Prior to processing of the image sequence, integer coordinates of grid nodes are set at which the velocity vectors should be estimated. Then, a small reference window is picked around each grid node in each frame. The size of the reference window along the x and y axes are Nx and Ny pix, respectively. Each reference window in the frame k is compared with the windows of the same shape and size displaced from the reference window in the frame *k* + 1. The displacements of reference windows along x and y coordinates are measured in pixels. Similarity between the windows is calculated using a functional. After the window displacements have been calculated at all grid nodes, vector field of velocity is calculated for each pair of successive frames. At each point of the grid, two components of velocity vector are estimated along x and y axes averaged over the time period equal to the time between the frames. If the time between frames is small in context of the considered problem, the obtained velocities can be considered instantaneous. To estimate the physical velocity of flow, dimensions in pixels should be converted to the metric system. For this purpose, targets with known dimensions were shot in the light sheet prior to measurements. In SIV measurements of velocity fields, frame-by-frame processing of high-speed videos in the near field of the cylinder was carried out. The framing frequency was 4000 Hz. The whole region of interest was divided into 20 × 20 pix windows, so the size of each pixel was 0.0414 mm. In the algorithm of velocity vector estimation, the window displacement was as follows: +15 and −2 pix along the coordinate x (in the direction of undisturbed flow velocity), +3 and −3 pix along the coordinate y (normal to the flow velocity). To eliminate major measurement errors, we employed two-stage filtering procedure. Vorticity was calculated using Stokes theorem with the approximation of velocity circulation using a first-order accuracy difference scheme.

## Ethics Statements

This material is the authors own original work, which has not been previously published elsewhere

## CRediT authorship contribution statement

**Andrei Miheev:** Investigation, Formal analysis, Writing – review & editing.

## Declaration of Competing Interest

The authors declare that they have no known competing financial interests or personal relationships that could have appeared to influence the work reported in this paper.

## Data Availability

Data on the influence of dynamical impact by mixing on characteristics of turbulent flows in circular pipes (Original data) (Mendeley Data).

## References

[1] Davletshin I.A., Mikheev A.N., Mikheev N.I., Shakirov R.R. (2020). Heat transfer and structure of pulsating flow behind a rib. Int. J. Heat Mass Transf..

[2] Molochnikov V.M., Mikheev N.I., Mikheev A.N., Paereliy A.A., Dushin N.S., Dushina O.A. (2019). SIV measurements of flow structure in the near wake of a circular cylinder at *Re*=3900. Fluid Dyn. Res..

[3] Mikheev A.N. (2018). Turbulent characteristics in the cylinder near wake estimated by SIV measurements. J. Phys..

[4] Mikheev N.I., Dushin N.S. (2017). Saushin I.I. Capabilities of optical SIV technique in measurements of flow velocity vector field dynamics. J. Phys..

[5] Dushin N.S., Mikheev N.I., Dushina O.A., Zaripov D.I., Aslaev A.K. (2017). Validation of SIV measurements of turbulent characteristics in the separation region. J. Phys..

[6] Molochnikov V.M., Kratirov D.V., Mikheev А.N. (2017). Pipe flow pattern downstream of local restrictions studied by an optical method. J. Phys.: Conf. Ser..

